# Oral health of 6–7 year-old children according to the Caries Assessment Spectrum and Treatment (CAST) index

**DOI:** 10.1186/s12903-018-0709-x

**Published:** 2019-01-17

**Authors:** Azadeh Babaei, Afsaneh Pakdaman, Hossein Hessari, Ahmad R. Shamshiri

**Affiliations:** 1 Community Oral Health Department, School of Dentistry, Tehran University of Medical Sciences, Postal code 1439955934 Tehran, Iran; 2 Research Center for Caries Prevention, Dentistry Research Institute, Tehran University of Medical Sciences, Postal code 1417614411 Tehran, Iran

**Keywords:** Dental caries, Child, Disease progression

## Abstract

**Background:**

The index of Caries Assessment Spectrum and Treatment (CAST) reveals a range of caries development from a non-cavitated status to advanced lesions. The aim of the present study was to explore the oral health status of 6- to 7-year-old children based on the CAST index in relation to oral health knowledge and background determinants.

**Methods:**

A multi-stage cluster random sampling method was applied and after ethical clearance, clinical examination was performed (Kappa = 0.89). The status of caries and oral hygiene was recorded according to the CAST index and OHI-Simplified (OHI-S) index, respectively. A self-administered questionnaire was used to collect the data of parental knowledge of oral health. SPSS version 22.0 was used for data analysis and *p*-value less than 0.05 were considered significant.

**Results:**

Seven hundred and thirty-nine children and their parents in 24 schools participated in this study (88%), of whom 48.6% were boys and the rest were girls. In permanent molars, a healthy status (code 0–2) was observed in 89.3–93.7% of the teeth. In primary molar teeth, dentinal lesions ranged from 25.3 to 31.2%, the prevalence of pulp involvement was between 2.9 and 10.5%, and less than 1% had abscess/fistula. Serious morbidity (codes 6 and 7) were more common in the first primary molars than the second ones. Multi-variable logistic regression analysis indicated that children with a low level of father’s education were 2.45 times more likely to have a CAST score of 3 and higher (95% CI 1.35–4.46, *p* = 0.003) compared to children whose fathers had academic education. For each one-unit increment of OHI_S, the likelihood of a CAST score 3 and higher in primary dentition increased by 1.77 times (OR = 1.77; 95% CI 1.08–2.93, *p* = 0.02).

**Conclusion:**

The consequences of dental caries including abscess and fistula were more prevalent in the first and second primary teeth. There was a significant correlation between a CAST score of 3 and higher with father’s education (as an indicator of social rank) and oral hygiene status. The CAST index is a useful and practical index in epidemiological surveys.

**Electronic supplementary material:**

The online version of this article (10.1186/s12903-018-0709-x) contains supplementary material, which is available to authorized users.

## Background

Several indices are used to describe the dental caries status at the national and community level covering a wide range of definitions for caries. The DMFT/dmft index as a World Health Organization criterion for measurement of dental caries has been widely used for decades in numerous epidemiological surveys [1]. However, this index does not reflect the consequences of untreated dental caries, including pain and infection. In recent years, a new concept of caries measurement describes caries from a non-cavitated level to a visible cavitation. Considering the dynamic nature of caries, it is important to use a measurement system that reflects non-cavitated lesions in order to apply caries-control measures. On the other hand, there is a need to report caries and its consequences such as infection, especially in developing countries where caries mostly remains untreated. Recent indices such as the ICDAS [2] and PUFA/pufa [3] have some limitations. The ICDAS index shows only caries before pulp involvement, and the PUFA/pufa index is used to investigate the severe conditions of caries without considering the non-cavitated status.

On this basis, Frencken et al., developed a new index of Caries Assessment Spectrum and Treatment (CAST) in 2011 to detect a range of caries statuses from a non-cavitated status to advanced lesions, which has been proved suitable for use in field surveys. In this index, dental caries is recorded using a wide range from sound to advanced enamel and dentine lesions in addition to the clinical consequences of caries including pulp involvement and abscess/fistula [4]. The CAST index is based on the visual-tactile examination and can be used in epidemiological surveys [5].

In high-risk communities, where caries is prevalent, it is important to report caries with more details. The national oral health survey of Iranian children in 2012 indicated a high level of caries in the primary dentition and the mean dmft/DMFT index was reported with 5.16/0.38 in 6-year-old children [6]. As dental caries increases with age, there is a need to detect non-cavitated lesions in early stages, especially in this age group. Children in the age of 6 to 7 are considered target group as the first permanent molar erupts in this age and if neglected, these teeth are vulnerable to caries.

The face and content validity of the CAST index have been tested and reported in the literature using the consensus method, according to the scoring of 15 senior epidemiologists from 15 countries over three rounds of assessments [7]. Since the development of the CAST index, many studies have used it to report the prevalence of dental caries in developed and developing countries. In a study by Baginska et al.*,* [5] in 2014, 7–8 year-old children were examined and the prevalence of caries was reported in primary molars. Advanced consequences of caries including cavitated dentine lesions, pulpal involvement, abscess/fistula, and missing teeth due to caries, were prevalent in the first and second primary molars. A strong correlation was found between the status of teeth on the right and left side of the oral cavity. Other studies conducted in Brazil, India, and Poland also reported the caries status using this index. However, no study in Iran has used this index to report a wide spectrum of dental caries in children.

The purpose of the present study was to explore the oral health status of 6- to 7-year-old children using the CAST index and Oral Health Index-Simplified (OHI-S) in relation to their background determinants and parental knowledge of oral health in Tehran, Iran.

## Methods

This study is the first report of a community-based trial in 6- to 7- year-old children in Tehran, Iran. Ethical clearance was sought from the Ethics Committee of Tehran University of Medical Sciences (IR.TUMS.REC.1394.1730). The protocol and objectives of the study was explained to subjects in a simple language and informed consent was obtained from parents (mothers) before the study.

### Sampling

A multi-stage cluster random sampling method was applied in different regions of Tehran according to the Ministry of Education districts. The sample size was adjusted according to the socio-economic status, considering affluent (1 to 8) and non-affluent areas (9 to 19) as strata. In each stratum, three districts were randomly selected and in each district, four schools were randomly chosen including two girl’s schools and two boy’s schools. Based on a pilot study, with the proportion of children with pulp involvement *P* = 0.3, α = 0.05, β = 0.2, a loss to follow up of 20%, and a design effect of 1.5 the sample size was calculated as 720 subjects with a power of 80%. Children were excluded from the study if they did not provide their parents’ consent, were not cooperative, had a dental emergency, or suffered from a systemic disease.

### Data collection

Data Collection consisted of two parts; A) collecting demographic and oral health information through a validated questionnaire [8] and B) clinical examination [9]. The questionnaires were delivered to children to be completed at home and the completed questionnaires were collected by the researcher (AB). To increase the response rate, a reminder was sent to parents and the questionnaires were collected after 1 week.

#### Questionnaire

After obtaining informed consent, a self-administered questionnaire was applied to collect data on socio-demographic and oral health knowledge. Demographic variables included age, gender, father’s education, mother’s education, birth order, and number of children in the household. The economic status was measured using the average living area in square meters per person (m^2^/p) as a proxy measure [10, 11]. This proxy measure was then categorized into two levels: less than 20 and equal or more than 20 square meters per person. In our study, years of education was used as a measure of social position. Education was categorized as illiterate, elementary school (1 to 5 years), middle school (6 to 8 years), high school or diploma (9 to 12 years), university degree (Associate: 2 years, Bachelor’s: 4 years, Master’s: 6 years, and Doctorate: 8 years).

The parents’ questionnaire contained 9 questions about oral health knowledge. The validity and reliability of this questionnaire were confirmed in previous studies [8, 12–15]. The questions were related to “microbial plaque” (2 questions; Tooth decay is caused by microbial plaque, Gum disease is caused by microbial plaque), “diet” (2 questions; Eating sweet food does not cause tooth decay, Restricting consumption of cookies, chocolate, candies, and other sugary snacks helps prevent dental caries), “oral hygiene behavior” (2 questions,; Brushing without toothpaste is enough for preventing dental caries, Regular tooth brushing helps prevent gum problems), “role of inheritance” (1 question; The main cause of tooth decay is inherited), “fluoride” (1 question; Fluoride is useful for teeth) and “mouthwash” (1 question; Rinsing with salt water or other kinds of mouth rinses is sufficient to clean teeth). The responses were recorded in a Likert scale from “completely agree” to “completely disagree” and were re-coded as true or false [8].

#### B) Clinical examination

Dental caries and oral hygiene status were regarded as outcome variables. In the school setting, 6- to 7- year-old children underwent oral examination. Dental caries examination was done using a disposable dental mirror and probe under artificial light by one examiner (AB) calibrated by an expert (AP). For calibration, 20 children [16] were examined by both examiners, AB and AP, considering a visual guide containing information on the CAST index and coding developed by Frencken et.al [9]. Consensus was achieved on each code after reviewing and discussing the codes, and inter-examiner reliability was further calculated (Kappa = 89.8).

**Fig. 1:**
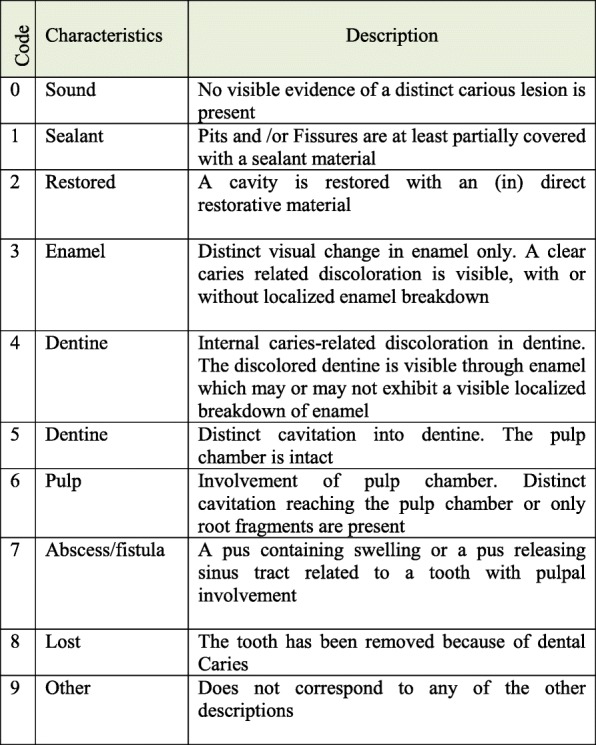
The explanation of codes in the CAST index

The CAST index scoring system is as follows: “0: sound”, “1: sealant”, “2: restoration”, “3: enamel lesions”, “4, 5: dentine lesions”, “6: pulp involvement”, “7: abscess/fistula”, “8: tooth loss”. If a situation did not match any codes from 0 to 8, a code 9 was assigned (Fig. 1). The codes 0–2, 3, 4–5, 6–7, and 8 were considered as “healthy”, “pre-morbidity”, “morbidity”, “serious morbidity”, and “mortality”, respectively [5]. The OHI-S index was applied to measure the oral hygiene status [17].

### Statistical analysis

The Statistical Package for Social Sciences (SPSS) version 22.0 was used for data analysis. Descriptive statistics, including the frequency distribution of subjects with different demographic characteristics, are used to present the data. The oral hygiene status of each subject was determined using the OHI-S index [17]. The frequency distribution of true/false answers for each knowledge question was analyzed, and the total score of knowledge (9 questions) was calculated. The maximum CAST code per subject was reported for clinical data.

Missing data imputation was done prior to regression analysis according to the EM (Expectation Maximization) algorithm. In this analysis, the Little’s MAR test was significant, so the data were considered Missing At Random (MAR). After imputing for missing values, logistic regression analysis was performed to assess the impact of independent variables on the unhealthy situation (CAST score > 3) as measured according to the CAST index. The significance level was set at 0.05.

## Results

Seven hundred and thirty-nine subjects returned the questionnaires (response rate = 88%). The socio-demographic characteristics of the study subjects including gender, father’s education, mother’s education, birth order, and number of children in the household and socio-economic status are presented in Table 1. Girls comprised 51.4% of the subjects and the rest were boys. As for the socio-economic status, 40.3% lived in an area of equal to or more than 20 square meters per person.

**Table 1:** Demographic characteristics of 6–7-year-old school children (*n* = 739)

	N	%
Gender		
Boy	359	48.6
Girl	380	51.4
Father’s Education		
Illiterate/Elementary school^a^/Middle school^b^	114	15.4
High school or Diploma^c^	272	36.8
Associate^d^/Bachelor’s/Master’s of science/Doctorate	307	41.5
Missing	46	6.2
Mother’s Education		
Illiterate/Elementary school^a^/Middle school^b^	97	13.1
High school Diploma^c^	305	41.3
Associate^d^/Bachelor’s /Master’s of science/Doctorate	292	39.5
Missing	45	6.1
Child birth order		
First	391	52.9
Second	195	26.4
Third	42	5.7
Forth & more	5	0.6
Missing	106	14.3
Number of children in the household		
1	252	34.1
2	349	47.2
3 and more	83	11.2
Missing	55	7.4
Socio-economic status^e^		
Less than 20	251	34
Equal or more than 20	298	40.3
Missing	190	25.7

^a^5 years

^b^6–8 years

^c^9–12 years

^d^2 years of academic education

^e^According to housing area (m^2^ per person) [11]

**Fig. 2:**
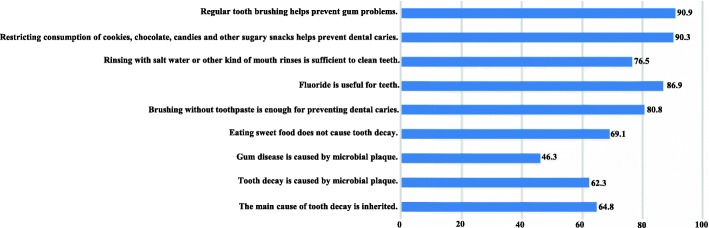
Distribution of respondents’ (parents’) correct answers (%) to questions regarding the knowledge of oral health (*n* = 739)

The parents’ knowledge of oral health is demonstrated in Fig. 2. The majority of the parents (90%) correctly identified that “Regular tooth brushing helps prevent gum problems” and “Restricting consumption of cookies, chocolate, candies, and other sugary snacks helps prevent dental caries”. Less than half of the parents (46.3%) responded correctly to “Gum disease is caused by microbial plaque”. More than 50% of the parents provided correct answers to the rest of questions on “Fluoride”, “Brushing with fluoridated toothpaste”, “Eating sweet food” “Microbial plaque” and “Inheritance of caries”.

Table 2 shows the percentage distribution of the caries status based on the CAST index in permanent and primary molar teeth. Of the first permanent molar teeth, the majority (82.6–90.7%) was scored as sound (code 0) and 2.3–4.4% had fissure sealant (code 1). In addition, 4.1–5.8% of these teeth had enamel lesions (code 3). Regarding the first permanent molar teeth, none of the subjects had pulp involvement, abscess/fistula, or tooth loss.

**Table 2:** Distribution of caries in primary and permanent molars of children according to the CAST index (*n* = 739)

	Sound	Sealant	Restoration	Enamel	Dentine		Pulp	Abscess /Fistula	Lost
	0	1	2	3	4	5	6	7	8
16	90.3	2.9	0.3	4.1	0.9	1.4	0	0	0
26	90.7	2.3	0.7	4.2	0.5	1.7	0	0	0
36	82.6	3.8	2.4	5.3	3	2.9	0	0	0
46	82.9	4.4	2	5.8	2	2.8	0	0	0
55	49.4	0.3	12.9	2.2	2.6	28.6	2.9	0.1	0.8
65	48.1	0.3	11.4	2.6	3.7	27.2	5.2	0.3	1.1
75	38.7	0.7	19.8	2.3	6.2	21.2	8.1	0.1	3
85	40.5	0.6	20.2	2.6	5.7	19.6	8.6	0	2.1
54	46.6	0	13.4	0.6	1.9	25.8	6.7	0.7	4.3
64	45.2	0	13.2	0.4	2.2	26.4	7.6	0.1	4.9
74	37.3	0	19	1	2	23.6	10.5	0.3	6.3
84	37.7	0	19.2	1.4	2.6	23.5	10.4	0	5.3

**Fig. 3:**
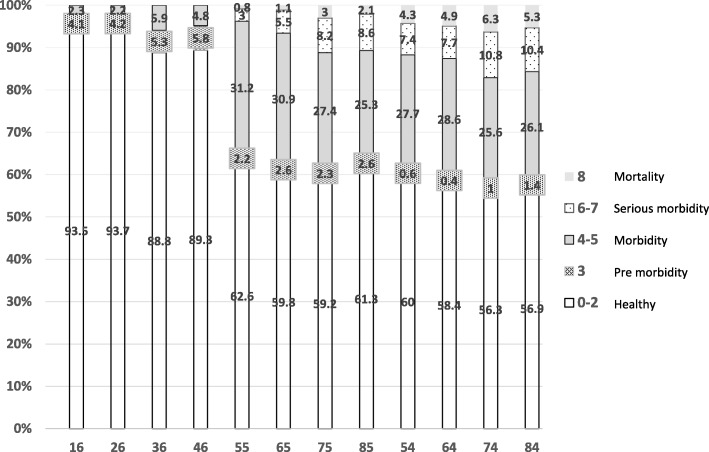
Distribution of the dental health status of the primary and permanent molars (healthy, pre-morbidity, morbidity, serious morbidity, mortality)

For the first primary molars, the prevalence of dentine caries was less than 30%, and pulp involvement and abscess/fistula were observed in 10.5 and 0.7%, respectively. The distribution of the lesions is demonstrated in Fig. 3. In the erupted first permanent molars, the healthy condition (code 0–2) ranged from 88.8 to 93.7%. Pre-morbidity and morbidity stages defined as enamel and dentine lesions (code 3, code 4–5) were observed in less than 6% of the teeth in the study subjects.

For the first and second primary molars, more than 50% were scored as healthy (code 0–2). Enamel caries (pre-morbidity) was observed in less than 3% of the teeth. In these teeth, dentine caries (code 4–5) was observed in 25.6–28.6% and 25.3 -31.2%, respectively. Serious morbidity as pulp involvement and abscess/fistula (codes 6–7) was seen in about 10% of the first primary molars and less than 10% of the second primary molar teeth. Less than 6% of the first and second primary molar teeth were lost due to caries.

In Table 3, the multi-variate logistic regression analysis showed that oral hygiene status and father’s education were associated with a CAST score of 3 or higher. The children with a lower level of father education (illiterate/elementary school/middle school) were 2.45 times more likely to have a CAST score of 3 or higher (95% CI 1.35–4.46, *p* < 0.003) compared to children whose fathers had an academic degree. In addition, children whose fathers had intermediate level of education (high school/diploma) were 1.55 times more likely to have a CAST score of 3 or higher (95% CI 1.06–2.28, *p* = 0.02) in comparison with the reference category (academic degree). For each one-unit increase of the OHI-S index, the likelihood of a CAST score of 3 or more increased by 1.77 times (OR = 1.77; 95% CI: 1.08–2.93, *p* = 0.02). Data provided in the Additional file 1 (Data sheet.xls).

**Table 3:** Logistic regression analysis of the association between having CAST score of > = 3 with demographic, knowledge and oral health related variables

	CAST> = 3		Unadjusted		Adjusted		
	n (%)		OR (95% CI)	P*	OR (95% CI)		P**
Gender							
Male	273 (79.1)		1.23 (0.86–1.74)	0.25			NS
Female	281 (75.5)		1				
Father’s Education							
Illiterate/Elementary school/Middle school	98 (86.7)		2.58 (1.42–4.66)	0.002	2.45 (1.35–4.46)		0.003
High school or Diploma	220 (80.0)		1.58 (1.08–2.31)	0.02	1.55 (1.06–2.28)		0.02
Associate/Bachelor’s/Master’s of science/Doctorate	236 (71.7)		1		1		
Mother’s Education							
Illiterate/Elementary school/Middle school	83 (85.6)		2.30 (1.24–4.27)	0.008			NS
High school or Diploma	247 (79.9)		1.58 (1.07–2.25)	0.02			NS
Associate/Bachelor’s/Master’s of science/Doctorate	224 (72.0)		1				
Child birth order							
2nd or more	288 (40.2)		1.33 (0.93–1.91)	0.12			NS
Single or 1st child	429 (59.8)		1				
Socio-economic status							
< 20	245 (81.1)		1.47 (1.03–2.12)	0.04			NS
≥ 20	309 (74.5)		1				
Supervised Children ‘s toothbrushing							
No	243 (78.6)		1.15 (0.81–1.64)	0.45			NS
Yes	311 (76.2)		1				
Knowledge (correct answer)							
≤ 8	591 (82.4)		1.53 (1–2.36)	0.05			NS
9	126 (17.6)		1				
OHIS			1.88 (1.15–3.09)	0.01	1.77 (1.08–2.93)		0.02

*p value of Unadjusted

**p value of adjusted

## Discussion

In this study, the prevalence of dental caries and its consequences was reported in 6–7 year-old children using the CAST index in relation to demographic and oral health variables and parents’ knowledge. In deciduous dentition, dentine caries was common in the first and second primary molar teeth and one in 10 children had pulp involvement, but only less than 1% of children had abscess/fistula. The majority of the permanent molar teeth was sound, and few teeth had enamel and dentine lesions although preventive care in the form of fissure sealant was uncommon. The children with poor oral hygiene and low father education were more likely to have a CAST score of 3 and more in primary molar teeth.

In our study, there was no association between gender and a caries score of 3 and more, which is in line with the results of a study by Gupta et al., [18] However, a significant relationship was observed between gender and dental caries in studied conducted by Kumar et al., [19] and Biria et al., [20]. Moreover, there was no association between being the only child or the first child and a caries score of 3 and more in the regression analysis, which is inconsistent with the results of a study by Folayan et al., that reported an association between caries and birth order [21].

Most parents had poor knowledge of the role of the microbial plaque in development of caries and gum disease. The information obtained showed that parents were not aware of the role of microorganisms in poor oral health, which might be due to the fact that the word ‘plaque’ is unfamiliar to parents. A previous local study by Gholami et al., investigating the adults’ knowledge of gum diseases and the role of “plaque” showed similar findings [22].

The prevalence of subjects with caries-free permanent molar teeth was high, which is in line with the results of a study by Dorenia et al., in Shimla. [23] but is in contrast to the findings of a study by Shyam et al., in 2017 [24] reporting a lower level of caries-free in the first permanent molars in India. Considering a recent guideline on fissure sealant, it is strongly recommended that the use of sealants should be considered in permanent molars with both sound occlusal surfaces and non-cavitated occlusal carious lesions in children and adolescents [25]. Considering the low level of fluoride in drinking tap water in Tehran (0.39 mg/L), it is important for children to receive preventive care [26].

In the present study, considering the fact that the first permanent molar teeth were newly erupted, the prevalence of receiving preventive care as fissure sealant was low. This finding was consistent with the results of study by Dorenia et al., reporting a low prevalence of children with fissure sealant in Indian children [23]. However, it is in contrast to the findings of Baginska et al., who reported that one-third of Polish children had fissure sealant in the first permanent molars [5].

The reason for this difference might be the fact that fissure sealant is considered an expensive preventive service. Preventive and therapeutic dental services are covered by insurance in Iranian rural areas and small cities but these services, including fissure sealant therapy, are not free of charge in urban areas [27]. On the other hand, this difference might reflect the fact that the level of awareness of parents on this issue was low and they did not feel the need for preventive care [28]. In polish children, the prevalence of restoration in deciduous teeth was higher compared to our subjects, indicating more access to dental services [5]. In our study, few permanent molar teeth had enamel lesions (premorbidity stage), which was less than other studies [5, 23].

In our study, the consequences of dental caries in the permanent dentition were not common and none of the subjects had pulp involvement, abscess, or fistula. Similarly, Baginska et al., reported scarce evidence of advanced caries as code 6 or more in permanent dentition [5]. However, Doneria et al., [23] reported a higher prevalence of pulp involvement in the first permanent molar compared to our participants.

The prevalence of enamel lesions in deciduous dentition was less than 5% in our study while it was more than 15% in the study by Doneria et al., [23]. In our study, a high prevalence of dentine caries was observed in the first and second primary molar teeth, which corroborates the fact that caries in deciduous teeth is prevalent in Iran [6]. Dentinal caries was more prevalent in the second deciduous molars compared to the first deciduous molars. Our findings of a high prevalence of caries in deciduous teeth is in agreement with the reported data on Polish and Indian children [5, 23].

In our study, more than a quarter of the children had distinct cavitation in primary molars dentition and about 10% had pulp involvement. A recent study [29] showed similar results in 6- to 7- year-old Polish children. The prevalence of cavitated dentine lesions (a CAST score of 5) and pulp involvement was high in the primary molars. This is consistent with the results of a study by Baginska et al.*,* [5] reporting a high prevalence of pulp involvement, abscess/fistula, and tooth loss in the first deciduous molars compared to the second deciduous molars.

A national oral health survey of Iranian children in 2015 showed a mean dmft index of 5.84 in 6-year-old children [6], but the burden of disease could not be estimated. Although the DMFT/dmft index is simple and easy to apply, it does not reflect the need for dental care as filling and decay has the same score in the total DMFT/dmft. Comparison of other indices such as ICDAS and PUFA/pufa with the CAST index, which present more details on the caries status shows that [30] although the ICDAS index provides detailed information, it is not applicable in field surveys since compressed air is needed to dry tooth surfaces [2].

On the other hand, the analysis of the data collected according to the ICDAS index is very complex and enamel lesions are classified into three levels [30]. In addition, the ICDAS index does not assess sealants, restorations, pulp involvement, and tooth loss [23] and hence its applicability in developing countries with high prevalence of caries is limited. The PUFA/pufa index reflects the consequences of caries including pulp involvement, ulceration, fistula, and abscess but it does not define non-cavitated carious lesions [3]. The advantage of using the CAST index is that it reports a full spectrum of dental caries from sound to pulp involvement, abscess/fistula, and tooth loss as severe consequences of caries. Therefore, the CAST index is suitable for identification of the need for preventive and curative care at the same time. It also may help policy-makers to allocate more resources to high-risk children in advanced stages of caries having pain and infection [31].

Many studies have shown a correlation between the caries prevalence and parents’ education. In our study, father’s education had a negative correlation with dental caries. Those children having father with no academic degree were more likely to have dental caries (CAST score 3 or higher). This finding is consistent with the results of a study by Kumar et al., in 2016 that showed both low education of parents and low socio-economic status increased caries in 12-year-old Indian schoolchildren [19]. The same finding was reported in Lithuanian children [32]. Also in our study, those children with poor oral hygiene as assessed according to OHI-S index were more likely to have CAST score of 3 or higher. The findings of Shabani et al., [33] showed a strong correlation between DMFT and OHI-S index in 10–15 years old children, which is in line with our study.

However, previous study [34] showed a higher prevalence of dental in children in the high and middle socio-economic class, which may be related to high sugar intake. We found no correlation between caries and socio-economic status, which might be due to the fact that the caries process is multi-factorial. Moreover, according to the local evidence, income information might be unreliable and people have more than one job at the same time. Therefore, we used the housing area per person for reporting the association between health and social status in Iran, which is a valid and reliable indicator [10]. However, using this index for reporting the socio-economic status instead of the income level makes comparison difficult.

### Strengths

One of the positive points of the present study was the high response rate of the parents. In addition, a representative sample was selected using a multistage random cluster sampling adjusted for the socio-economic status and a valid questionnaire was administered. Therefore, the results can be used by local policymakers to improve the children’s oral health. Moreover, we applied the CAST index, a validated index that provides more accurate and comprehensive information compared to other indices in order to detect a range of lesions including non-cavitated and advanced lesions. We used a visual aid including the details of the CAST index during calibration and dental examination to reduce the risk of misclassification.

### Limitations

The CAST index was used in this study, which is more complicated than the DMFT/dmft index and therefore the process of calibration was time-consuming and more time was needed to reach consensus; however, using a visual aid was helpful. There were some limitations in earning the contribution of the parents to complete the questionnaire and obtaining their consent before the survey. In order to increase the response rate, one of the researchers (AB) sent a reminder to parents. Our analyses were based on the information provided by respondents. However, according to the missing data analysis, non-respondents were randomly distributed in the sample and therefore, we assume that there was no specific difference between subjects who responded and those who did not.

## Conclusion

In summary, this study was conducted to evaluate the prevalence of dental caries using the CAST index. The first permanent molar teeth were mainly sound and enamel caries existed on few teeth. Preventive services in the form of fissure sealant were scarce. In the primary molar teeth, the consequences of caries including pulp involvement were common (one in ten teeth). Participants with a poor oral hygiene whose fathers lacked academic education were more likely to have a CAST score of 3 or more.

## Additional file

Additional file 1: Data sheet. (XLS 220 kb)

## Abbreviations

CAST: Caries Assessment Spectrum and Treatment
DMFT/dmft: Decayed (D/d), Missing (M/m), Filled (F/f) Tooth
EM: Expectation Maximization
ICDAS: International Caries Detection and Assessment System (dentistry)
MAR: Missing At Random
OHI-S: Oral Health Index-Simplified
PUFA/pufa: Pulpal involvement (P/p), ulceration (U/u), fistula (F/f) and abscess (A/a)
SPSS: Statistical Software for Social Sciences
